# Challenges and Insights: Cervical Spine Injuries in Children with Traumatic Brain Injury

**DOI:** 10.3390/children11070809

**Published:** 2024-07-02

**Authors:** Hannah K. Weiss, Richard C. E. Anderson

**Affiliations:** Department of Neurosurgery, NYU Langone Health, New York, NY 10016, USA

**Keywords:** cervical spine injury, trauma, pediatric, traumatic brain injury

## Abstract

Cervical spine injuries (CSIs) in pediatric patients with traumatic brain injury (TBI) pose unique diagnostic and management challenges. Current studies on the intricate overlap between pediatric TBI and CSI are limited. This paper explores the existing literature as well as the epidemiology, mechanisms of injury, diagnostic criteria, treatment strategies, and outcomes associated with CSI in pediatric TBI patients.

## 1. Introduction

Traumatic brain injury (TBI) represents a major cause of disability worldwide. Globally, approximately 69 million individuals experience a TBI each year [1]. TBI in pediatric patients is a major public health concern, as TBI often results in substantial long-term consequences for both the patient and their family [2]. Many TBIs are caused by high-energy accidents, often leading to polytrauma injuries. Cervical spine injury (CSI) can exacerbate the morbidity associated with TBI, further affecting long-term outcomes in pediatric TBI patients [3].

Despite the global prevalence of pediatric TBI, there is limited information specifically addressing TBI patients and associated cervical spine injuries [3]. Although pediatric spine trauma is rare, given the major impact that TBI has in young populations, the association of related spine injuries remains relevant and is crucial to study in order to develop improved diagnostic and management strategies.

The aim of this paper is to synthesize current understanding and clinical practices related to the management of cervical spine injuries in pediatric TBI patients. We seek to identify studies that have analyzed the relationship between spinal injuries and TBI in pediatric patients and summarize common findings and outcomes to guide future research and clinical practice.

## 2. Epidemiology of Cervical Spine Injuries in Pediatric TBI

The incidence and type of cervical spine injuries in children vary by age, mechanism of injury, and severity of the impact [4]. The developing pediatric spine has unfused synchondroses, incomplete ossification centers, and ligamentous hypermobility, thus making it more flexible than the adult spine [5]. The increased flexibility effect is twofold: the developing spine can endure more movement without damage to the spinal cord; however, the pediatric spine provides less protection in the setting of major spinal trauma [5]. In addition, children are more susceptible to cervical injuries due to their proportionally larger head size and under-developed neck musculature. The most unstable portion of the developing spine is the upper cervical spine, and as spinal maturity progresses, spinal injuries below the level of the cervical spine become increasingly common [6].

Traumatic brain injury in older pediatric patients is most often associated with motor vehicle accidents (MVAs) and other high-force/high-impact scenarios [7,8]. However, for younger patients suffering from TBI, abusive head trauma and non-motor vehicle crash injuries are more common [3]. Similarly, the etiology of cervical spine injury is often associated with high-impact trauma and varies by age: children under the age of nine are at higher risk of cervical spine injury secondary to falls and pedestrian–automobile accidents, while teenagers tend to be at higher risk for cervical spine injury secondary to motor vehicle accidents [6,9]. A retrospective study of young children (<2 years old) with non-motor vehicle crash-associated TBI found that patients with non-accidental head trauma had the highest rate of CSI [3].

A recent study by Bennet et al. studied hospitalized pediatric TBI patients and found that CSI was present in 1% [4] of patients [10]. A similar rate of injury has been found in pediatric blunt trauma cohort studies [11,12,13]. In cohorts including only severely injured pediatric trauma patients, the rate of CSI is as high as 9% [14]. Cervical spine injury in pediatric TBI patients has been shown to be associated with increased patient age, motor- vehicle accidents, and overall injury severity [10]. In addition, pediatric patients with more severe TBI are more likely to have CSI [3,10]. In a 2013 retrospective review of severely injured pediatric trauma patients, patients with CSI were more likely to have TBI and to be on a ventilator [14].

## 3. Mechanisms and Patterns of Injury

Cervical spine injuries, similar to TBI, in pediatric patients often result from high-energy trauma such as motor vehicle accidents, falls from standing (especially ages 2–9), falls from substantial heights, or sports-related impacts [11,15]. The biomechanical forces involved—flexion, extension, rotation, and compression—can lead to a range of injury types from simple soft tissue injuries to complex fractures and dislocations.

The four major categories of spinal injury patterns in pediatric patients include fracture with subluxation, fracture without subluxation, subluxation without fracture, and spinal cord injury without radiographic abnormality (SCIWORA). In the pediatric TBI cohort studied by Bennet et al., the most common spine injury was spinal column fracture without cord injury, whereas in the retrospective cohort of <2-year-old non-MVA TBI the most common injury was cervical spine extra-axial hemorrhage [3,10]. The upper cervical spine is more likely to be injured in young patients, and as the spine matures, subaxial injuries increase in frequency [3,10].

The most common craniocervical junction injuries that result from high-impact trauma and may be associated with TBI include atlanto-occipital dislocation (AOD), Jefferson fracture, translational atlantoaxial subluxation, odontoid fractures, os odontoideum, and Hangman’s fracture.

Atlanto-occipital dislocation is a high-risk, often fatal, ligamentous injury that results from hyperextension, leading to disruption of tectorial membranes and/or alar ligaments, causing injury to the O-C1 joint capsules. Children with AOD often present with severe neurologic deficits [16]. The mechanism of injury typically involves pedestrians struck by motor vehicles [17]. CT scan will reveal widening of the occipital condyle-C1 joint interval > 4 mm [16]. Suspicion of vertebral artery dissection should also remain high if clinically relevant symptoms are present. Treatment involves immediate immobilization and stabilization, with occipital-cervical fusion often required once medically stable.

**Figure 1:**
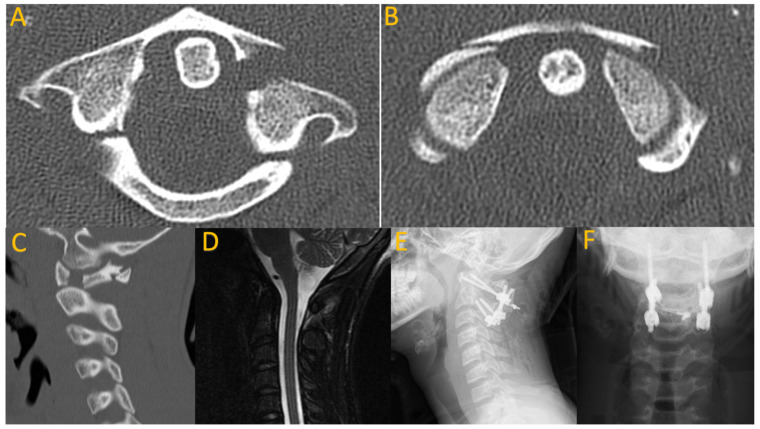
A 16-year-old male who presented with neck pain after diving into shallow water in the ocean, hitting a sand bar. He was neurologically intact. Axial (**A**,**B**) and sagittal (**C**) CT imaging reveals a four-part burst fracture of C1. Sagittal MRI STIR sequence suggests ligamentous injury (**D**). He underwent posterior C1–C2 fusion with instrumentation, wiring, and allograft (**E**,**F**).

Jefferson fractures commonly result from falls and motor vehicle accidents, and involve a four-part burst fracture of C1. They are rarely seen in young children given incomplete ossification and presence of cartilage helping to decrease overall transmitted force [18]. Jefferson fractures may be associated with injury of the transverse ligament and/or associated C2 fracture. Diagnosis can be made using plain radiographs or CT; transverse ligament integrity can be assessed utilizing the Rule of Spence (sum of overhang of bilateral C1 lateral masses on C2 lateral masses > 7 mm) as well as MRI. Treatment typically involves external immobilization as long as the transverse ligament remains intact, with halo immobilization or C1–2 instrumentation and fusion if ligamentous instability exists (Figure 1) [19].

Translational atlantoaxial subluxation is a rare type of CSI in pediatric patients and involves compression of the spinal cord between the odontoid process and the posterior ring of C1. When secondary to a traumatic cause, these injuries often occur due to high-energy trauma such as pedestrian–motor vehicle accidents [4,20]. Diagnosis involves recognition of increased atlantodental interval (ADI) on lateral X-ray or sagittal CT (>5 mm in children < 8 years old; >3 mm in older children and adults) [21]. Treatment typically involves posterior C1–2 instrumentation and fusion.

**Figure 2:**
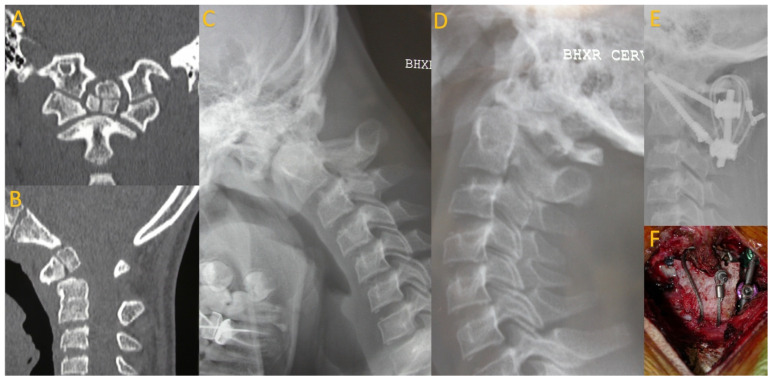
A 16-year-old boxer who presented after developing transient paraplegia while boxing. CT imaging revealed os odontoideum (**A**,**B**) with hypermobility on flexion and extension cervical X-rays (**C**,**D**). He underwent posterior C1–2 fusion with instrumentation, wiring, and allograft (**E**,**F**).

Odontoid fractures, although rare in pediatric patients, can occur at the dentocentral synchondrosis, generally in younger patients (as the dentocentral synchondrosis fully fuses between 9 and 10 years) [22]. Anterior angulation or displacement of the odontoid process can be seen on lateral X-rays. These fractures are most often treated with external immobilization [23]. Os odontoideum involves the superior portion of the odontoid process being separate from the rest, often considered to be congenital, but increasing evidence suggests it is associated with chronic nonunion or an odontoid fracture [23,24]. Os odontoideum may be especially relevant in pediatric patients with a remote history of TBI, and often requires C1–2 instrumentation and fusion to avoid further neurologic complications (Figure 2) [24].

Hangman’s fractures result from hyperextension injuries, including whiplash from motor vehicle accidents or collision during contact sports. Imaging will reveal varying degrees of avulsion of the C2 neural arch and anterolisthesis of the C2 vertebral body. These injuries can typically be managed conservatively with immobilization in a rigid collar. If there is >3 mm displacement of C2 on C3, halo immobilization is the preferred treatment, but surgical stabilization may be required [25].

**Figure 3:**
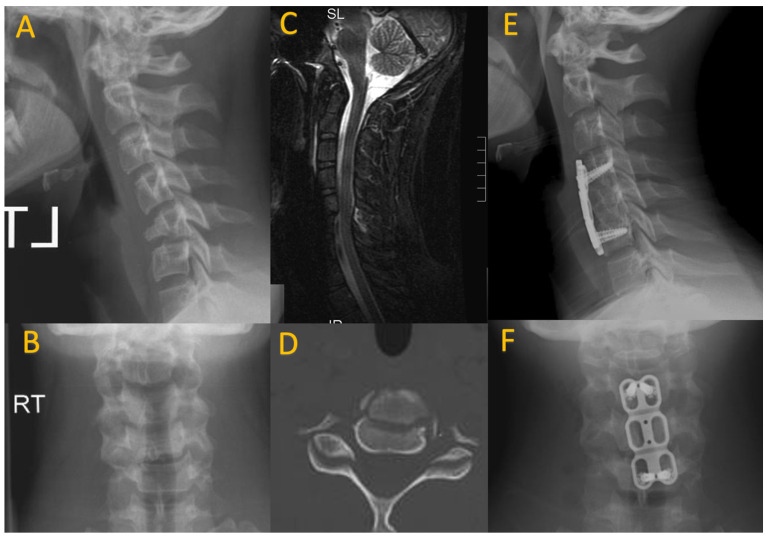
A 14-year-old male who presented after hitting a tree while snowboarding. On presentation he complained of transient weakness and persistent neck pain. He was found to have a C5 teardrop fracture on sagittal and AP cervical spine X-rays (**A**,**B**). There was interspinous ligament injury noted on the sagittal MRI STIR sequence (**C**). CT axial imaging again demonstrated the anterior column fracture (**D**). He underwent C5 corpectomy with anterior C4–6 instrumentation and allograft interbody strut (**E**,**F**).

Subaxial cervical spine injuries include ligamentous injuries, anterior column injuries, posterior column injuries, and combined anterior and posterior column injuries. Ligamentous injuries can be diagnosed using dynamic X-rays. Horizontal displacement of one vertebral body on another in the young pediatric spine can be considered normal when up to 4 mm; cervical vertebral subluxation of greater than 4.5 mm should raise concern for ligamentous instability [26]. Anterior column injuries include teardrop fractures, longitudinal fractures, wedge compression fractures, and burst fractures (Figure 3). Posterior column injuries involve injuries to the facets, lamina, pedicles, or spinous process. Combined anterior and posterior column injuries, involving both ligamentous and osseous injury, are less common in the pediatric population, but when present are highly unstable and often secondary to a high-energy mechanism [27].

## 4. Diagnostic Challenges

Diagnosing CSI in the context of pediatric TBI involves multiple modalities, from physical examination to advanced imaging techniques. The nuances of interpreting radiological findings in children, who may have normal anatomical variants that appear pathological, add complexity to the diagnostic process [5].

Children with mild TBI and low suspicion for CSI may qualify for physical exam alone in evaluating for cervical spine injury [28]. The National Emergency X-Radiography Utilization Study (NEXUS) criteria are now validated in pediatric populations and can help determine the risk of possible CSI and need for further imaging; if the patient does not have midline cervical tenderness, evidence of intoxication, altered mental status, focal neurologic deficit, or painful distracting injury, they are considered low-risk and can avoid further imaging if there is a benign clinical exam. A retrospective study of pediatric TBI patients’ workup in the emergency department revealed that the proportion of pediatric TBI patients receiving no cervical spine imaging, and having obtained “clinical clearance”, has increased in recent years, likely secondary to initiatives such as the NEXUS criteria [10]. Even the youngest noncommunicative patients aged 0–3 years have been shown to be able to be “clinically cleared” with a benign clinical examination and normal plain X-rays [29].

On the contrary, a pediatric patient that has suffered from a severe TBI may not be able to participate in a neurologic exam or convey neck pain [28]. In these cases, it is critical to obtain imaging to rule out cervical injury, which can be missed with distracting injuries such as a severe TBI. A retrospective study of the Pennsylvania Trauma Outcomes Study database focused on TBI in patients with Glasgow Coma Scale (GCS) < 8; the analysis did not find any clear rule to safely dismiss advanced imaging workup in comatose patients [30]. Even though the rate of CSI in pediatric patients remains low, in patients with low GCS, stabilization in a cervical collar until imaging is obtained is critical [31]. The Pediatric Cervical Spine Clearance Working Group published a study in 2019 that applied the Delphi method with the aim of establishing a consensus on cervical spine clearance in pediatric patients who had experienced blunt trauma [28]. This multidisciplinary team, consisting of pediatric orthopedic surgeons, pediatric neurosurgeons, pediatric emergency medicine physicians, and radiologists established an algorithm based on GCS score to help establish a clear consensus on pediatric cervical spine clearance in blunt trauma patients. The algorithm and consensus conclude that patients with GCS 14–15 that answer “no” when asked about neck pain, difficulty with neck movement, or history of focal neurologic deficit, as well the absence of abnormal head position, midline neck tenderness, limited range of motion, inability to focus due to distracting injuries, or visible substantial injuries to other areas of the body on exam, history and exam for clearance will suffice [28]. In patients who answer “yes” to having neck pain, difficulty with neck movement, or history of focal neurologic deficit, or have any of the above concerning findings on exam, the recommendation is to obtain plain radiographs. If the radiograph is normal, a cervical collar can be obtained once physical exam findings are normal. However, if the radiograph is abnormal, or if the mechanism of injury involved a high-risk mechanism, then the recommendation is to maintain the cervical collar and obtain a formal spine consult. For patients with GCS 9–13, it is recommended that plain radiographs would suffice if the patient was expected to return to GCS 14–15. However, if not expected to return to baseline neurologic exam, or if GCS < 8, then CT of the cervical spine is recommended [28].

In recent years, the utilization of CT imaging for “clearance” of the cervical collar has been on the rise in both adults and children [10]. The exposure to radiation during CT is a significant factor to consider when deciding whether to pursue CT imaging in pediatric TBI patients. Pediatric patients are at higher risk for radiation given their longer life expectancy and increased radiosensitivity [10]. In recent years, the utilization of MR imaging has also increased [10]. The benefit of MR imaging is the sparing of ionizing radiation, as well as the ability to reveal ligamentous injury or spinal cord injury in patients who are altered from a TBI. The obvious disadvantage, however, is the expense and time needed to complete the study, often requiring sedation for young patients. The utilization of X-rays of the cervical spine has been found to be decreasing over recent years [10].

There is significant variation across hospitals regarding initial workup for cervical injury in pediatric TBI patients, and a retrospective cohort study revealed that particularly the use of CT imaging varies significantly between hospitals [10]. Children receiving care at level 1 trauma centers received cervical spine imaging less often, perhaps due to established hospital-wide policies encouraging clinical clearance and utilization of the NEXUS criteria when appropriate [10].

## 5. Management Strategies

The management of CSI in pediatric TBI patients is multifaceted, most often involving conservative management, but in the setting of ligamentous instability, unstable fractures, or neurologic injury, acute decompression and surgical stabilization may be necessary. Treatment decisions are guided by the type and severity of the injury [4]. In the setting of severe TBI, the risks and benefits of cervical stabilization need to be fully evaluated and the surgical team may need to delay surgical intervention until the patient is deemed stable for surgery. Previous studies have revealed that pediatric patients undergoing extracranial surgery, such as spine surgery, within 72 h of presenting with moderate to severe TBI are at higher risk of intraoperative secondary insults [32]. These secondary insults were found to be common and associated with intraoperative cerebral hypotension and hypoxia [32]. In cases of moderate to severe TBI, special considerations to avoid secondary intraoperative insult as well as monitoring intracranial pressure should be considered.

## 6. Prognosis and Outcomes

Recovery from cervical spine injuries in children with TBI can vary widely, from complete recovery to permanent impairment. Factors influencing outcomes include the initial severity of the injury, the presence of associated injuries, and the timeliness and appropriateness of the treatment received. Mortality has been shown to be increased in TBI patients with concurrent CSI [10].

In addition, the timeliness of ruling out CSI has also been shown to be important to avoid cervical collar-related pressure injuries in pediatric patients. A retrospective review of severely injured pediatric trauma patients found that 10% of patients experienced cervical collar complications such as pressure injuries [14]. Cervical collar complications were more common in patients with lower GCS and longer length of stay and length of time that elapsed prior to cervical collar clearance [14].

## 7. Discussion

The inter-relationship between TBI and CSI in pediatric patients presents complex challenges for diagnosis and management. The high global incidence of TBI among children, in addition to the variable and potentially severe nature of associated cervical spine injuries, underscores the need for guidelines for pediatric CSI workup and management. As indicated by the studies reviewed, pediatric patients have unique anatomical and physiological factors that alter the common injury patterns typically seen in adults.

Despite advancements in imaging techniques and diagnostic criteria, including the establishment of the NEXUS criteria and the validation in pediatric populations, significant challenges remain in the diagnosis of CSI in pediatric TBI patients. The balance between adequate, timely diagnosis and reducing unnecessary radiation exposure is a concern particularly relevant to the pediatric population. This is compounded by the need for sedation and the overall time needed for MR imaging, a factor that is especially relevant in patients with severe TBI or other polytrauma causing clinical instability.

Management strategies, although often involving conservative measures, are also complicated in the TBI patient population. The decision-making process regarding the timing and urgency of surgical stabilization, when indicated, is complicated by the neurologic injury and the risks of undergoing surgery while also needing to maintain measures to closely monitor intracranial pressure and neurologic decline.

## 8. Conclusions

This review highlights the intricate link between pediatric TBI and CSI, demonstrating that despite considerable research in pediatric TBI globally as well as cervical spine injury patterns in pediatric patients, gaps in the literature persist when looking specifically at the association between pediatric TBI and CSI. Pediatric patients with CSI and concurrent TBI have unique clinical factors that need to be considered when planning diagnostic workup and management of CSI. Future efforts focusing on the standardization of CSI workup in TBI patients and the utilization of existing criteria such as the NEXUS criteria and establishing cervical spine clearance protocols might enable less variation in workup between different patient care settings, decreased radiation exposure, and improvement in timely diagnosis. Efforts to standardize treatment protocols across healthcare facilities could potentially improve outcomes by ensuring that all patients receive evidence-based care.

Ultimately, enhancing our understanding of the epidemiology, mechanisms, and outcomes of CSI in pediatric TBI patients will require ongoing multidisciplinary research. These future research efforts will both improve immediate clinical care as well as allow for improved preventive measures and public health policies aimed at reducing the incidence and severity of these injuries among children worldwide.

## Data Availability

The original contributions presented in the study are included in the article, further inquiries can be directed to the corresponding author.

## References

[1] Dewan M.C., Rattani A., Gupta S., Baticulon R.E., Hung Y.-C., Punchak M., Agrawal A., Adeleye A.O., Shrime M.G., Rubiano A.M. (2018). Estimating the global incidence of traumatic brain injury. J. Neurosurg..

[2] Dewan M.C., Mummareddy N., Wellons J.C., Bonfield C.M. (2016). Epidemiology of Global Pediatric Traumatic Brain Injury: Qualitative Review. World Neurosurg..

[3] Henry M.K., French B., Feudtner C., Zonfrillo M.R., Lindberg D.M., Anderst J.D., Berger R.P., Wood J.N. (2021). Cervical Spine Imaging and Injuries in Young Children With Non-Motor Vehicle Crash-Associated Traumatic Brain Injury. Pediatr. Emerg. Care.

[4] McGrory B.J., Klassen R.A., Chao E.Y., Staeheli J.W., Weaver A.L. (1993). Acute fractures and dislocations of the cervical spine in children and adolescents. J. Bone Jt. Surg. Am..

[5] Fesmire F.M., Luten R.C. (1989). The pediatric cervical spine: Developmental anatomy and clinical aspects. J. Emerg. Med..

[6] Hadley M.N., Zabramski J.M., Browner C.M., Rekate H., Sonntag V.K. (1988). Pediatric spinal trauma. Review of 122 cases of spinal cord and vertebral column injuries. J. Neurosurg..

[7] Daniels D.J., Clarke M.J., Puffer R., Luo T.D., McIntosh A.L., Wetjen N.M. (2015). High occurrence of head and spine injuries in the pediatric population following motocross accidents. J. Neurosurg. Pediatr..

[8] Injury Prevention & Control: Traumatic Brain Injury & Concussion Percent Distributions of TBI-Related Hospitalizations by Age Group and Injury Mechanism—United States, 2006–2010. https://www.cdc.gov/traumaticbraininjury/data/dist_hosp.html.

[9] Leventhal J.M., Martin K.D., Asnes A.G. (2010). Fractures and traumatic brain injuries: Abuse versus accidents in a US database of hospitalized children. Pediatrics.

[10] Bennett T.D., Bratton S.L., Riva-Cambrin J., Scaife E.R., Nance M.L., Prince J.S., Wilkes J., Keenan H.T. (2015). Cervical spine imaging in hospitalized children with traumatic brain injury. Pediatr. Emerg. Care.

[11] Polk-Williams A., Carr B.G., Blinman T.A., Masiakos P.T., Wiebe D.J., Nance M.L. (2008). Cervical spine injury in young children: A National Trauma Data Bank review. J. Pediatr. Surg..

[12] Kokoska E.R., Keller M.S., Rallo M.C., Weber T.R. (2001). Characteristics of pediatric cervical spine injuries. J. Pediatr. Surg..

[13] Patel J.C., Tepas J.J., Mollitt D.L., Pieper P. (2001). Pediatric cervical spine injuries: Defining the disease. J. Pediatr. Surg..

[14] Chan M., Al-Buali W., Charyk Stewart T., Singh R.N., Kornecki A., Seabrook J.A., Fraser D.D. (2013). Cervical spine injuries and collar complications in severely injured paediatric trauma patients. Spinal Cord.

[15] Cirak B., Ziegfeld S., Knight V.M., Chang D., Avellino A.M., Paidas C.N. (2004). Spinal injuries in children. J. Pediatr. Surg..

[16] Dublin A.B., Marks W.M., Weinstock D., Newton T.H. (1980). Traumatic dislocation of the atlanto-occipital articulation (AOA) with short-term survival. With a radiographic method of measuring the AOA. J. Neurosurg..

[17] Astur N., Klimo P.J., Sawyer J.R., Kelly D.M., Muhlbauer M.S., Warner W.C.J. (2013). Traumatic atlanto-occipital dislocation in children: Evaluation, treatment, and outcomes. J. Bone Jt. Surg. Am..

[18] Judd D.B., Liem L.K., Petermann G. (2000). Pediatric atlas fracture: A case of fracture through a synchondrosis and review of the literature. Neurosurgery.

[19] Korinth M.C., Kapser A., Weinzierl M.R. (2007). Jefferson fracture in a child–illustrative case report. Pediatr. Neurosurg..

[20] Bohn D., Armstrong D., Becker L., Humphreys R. (1990). Cervical spine injuries in children. J. Trauma.

[21] Sherk H.H., Schut L., Lane J.M. (1976). Fractures and dislocations of the cervical spine in children. Orthop. Clin. N. Am..

[22] Gebauer M., Lohse C., Barvencik F., Pogoda P., Rueger J.M., Püschel K., Amling M. (2006). Subdental synchondrosis and anatomy of the axis in aging: A histomorphometric study on 30 autopsy cases. Eur. Spine J. Off. Publ. Eur. Spine Soc. Eur. Spinal Deform. Soc. Eur. Sect. Cerv. Spine Res. Soc..

[23] O’Brien W.T.S., Shen P., Lee P. (2015). The Dens: Normal Development, Developmental Variants and Anomalies, and Traumatic Injuries. J. Clin. Imaging Sci..

[24] Klimo P.J., Kan P., Rao G., Apfelbaum R., Brockmeyer D. (2008). Os odontoideum: Presentation, diagnosis, and treatment in a series of 78 patients. J. Neurosurg. Spine.

[25] Montalbano M., Fisahn C., Loukas M., Oskouian R.J., Chapman J.R., Tubbs R.S. (2017). Pediatric Hangman’s Fracture: A Comprehensive Review. Pediatr. Neurosurg..

[26] SULLIVAN C.R., BRUWER A.J., HARRIS L.E. (1958). Hypermobility of the cervical spine in children; a pitfall in the diagnosis of cervical dislocation. Am. J. Surg..

[27] Gigliotti M.J., Farou N., Salyvia S., Kelleher J., Rizk E. (2021). Cervical Pediatric Spine Trauma Managed With Open Spinal Fixation and Instrumentation and a Review of the Literature. Cureus.

[28] Herman M.J., Brown K.O., Sponseller P.D., Phillips J.H., Petrucelli P.M., Parikh D.J., Mody K.S., Leonard J.C., Moront M., Brockmeyer D.L. (2019). Pediatric Cervical Spine Clearance: A Consensus Statement and Algorithm from the Pediatric Cervical Spine Clearance Working Group. J. Bone Jt. Surg. Am..

[29] Anderson R.C.E., Kan P., Vanaman M., Rubsam J., Hansen K.W., Scaife E.R., Brockmeyer D.L. (2010). Utility of a cervical spine clearance protocol after trauma in children between 0 and 3 years of age. J. Neurosurg. Pediatr..

[30] Piatt J.H. (2006). Detected and overlooked cervical spine injury in comatose victims of trauma: Report from the Pennsylvania Trauma Outcomes Study. J. Neurosurg. Spine SPI.

[31] Arbuthnot M.K., Mooney D.P., Glenn I.C. (2017). Head and Cervical Spine Evaluation for the Pediatric Surgeon. Surg. Clin. N. Am..

[32] Fujita Y., Algarra N.N., Vavilala M.S., Prathep S., Prapruettham S., Sharma D. (2014). Intraoperative secondary insults during extracranial surgery in children with traumatic brain injury. Childs Nerv. Syst. ChNS Off. J. Int. Soc. Pediatr. Neurosurg..

